# Dual-metal sites enable conductive metal–organic frameworks with extraordinary high capacitance for transparent energy storage devices†

**DOI:** 10.1039/d5sc01584g

**Published:** 2025-04-15

**Authors:** Cui-e Zhao, Shuaikang Wang, Shaoqiang Chen, Bin Han, Shouhao Wan, Qijia Bai, Mingao Hu, Fangyuan Kang, Ruiqing Liu, Jiahui Li, Yanwen Ma, Qichun Zhang

**Affiliations:** a State Key Laboratory of Flexible Electronics (LoFE) & Institute of Advanced Materials (IAM), Nanjing University of Posts and Telecommunications 9 Wenyuan Road Nanjing Jiangsu 210023 China; b Department of Materials Science and Engineering, City University of Hong Kong Hong Kong SAR 999077 P. R. China qiczhang@cityu.edu.hk; c Suzhou Vocational Institute of Industrial Technology 1 Zhineng Avenue, Suzhou International Education Park Suzhou 215104 China

## Abstract

Two-dimensional (2D) conductive metal–organic frameworks (c-MOFs) with intrinsic electrical conductivity and framework structure have been considered promising electrode materials for flexible and transparent energy storage devices. However, balancing electrochemical properties and optical transmittance remains challenging. To address this issue, a strategy of dual-metal-sites 2D c-MOFs is proposed to expand 2D Cu-MOF to nanorod-combined 2D CuNi-HHTP (HHTP = 2, 3, 6, 7, 10, 11-hexahydroxy-triphenylene) with improved ion and charge transport to redox species for Faraday reactions in micro-supercapacitors. Density functional theory calculations reveal that the incorporation of Ni can optimize the insertion of pseudocapacitive cations (K^+^) on dual-metal sites, significantly enhancing electron transfer during the charge-storage process. Furthermore, a facile laser-scribing technique is adopted for the fabrication of the interdigital architecture, serving as a transparent platform with exceptional optoelectronic properties. As a result, the CuNi-HHTP MSC exhibits high optical transmittance (over 80%), ultrahigh areal capacitance (28.94 mF cm^−2^), high energy density (1.45 μW cm^−2^), high power density (61.38 mW cm^−2^) and decent cycle stability (over 5000 cycles). This work offers a means of rationally designing 2D c-MOFs for the advancement of flexible transparent portable electronics.

## Abstract 2

Dual-metal-sites conductive metal–organic-frameworks (c-MOFs) CuNi-HHTP enables effectiveions and charge transport, realizing high optical transmittance and large areal capacitance in interdigital transparent micro-supercapacitor (MSC).

## Graphical abstract

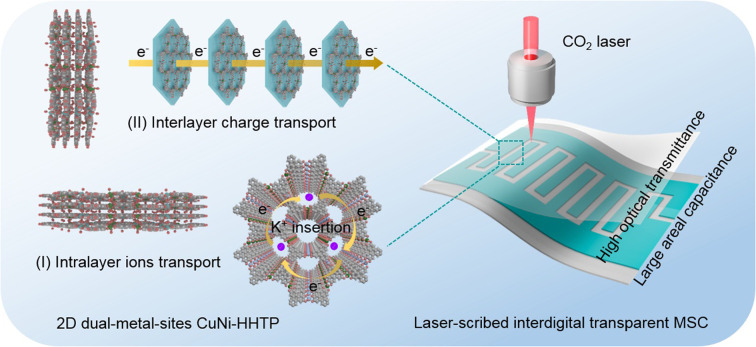

## Introduction

With the rise of the Internet of Things, developing transparent energy storage devices is extremely urgent, to meet the power requirements of the next generation of electronics.^1–3^ Flexible and transparent micro-supercapacitors (MSCs) have drawn much attention due to their advantages in terms of optical transparency, mechanical flexibility, light weight, and high power density.^4–7^ In particular, in-plane MSCs can be directly integrated with smart devices, and have opened up entirely new applications in wearable equipment,^8^ portable devices,^9^ intelligent sensors, *etc*.^10^ As one key component of MSCs, transparent electrodes with excellent optoelectronic properties are particularly crucial to match to portable electronics. Nonetheless, it is very challenging to balance the optical transparency and the volumetric capacitance simultaneously in one device.

In recent years, substantial research efforts have been made for finding high-volumetric electrode materials.^11–15^ Intrinsically conductive metal–organic frameworks (c-MOFs) have emerged as an important class of electrode materials for electrochemical energy storage, featuring effective π–π conjugation, diverse topological structures, and molecular-level redox-active sites.^16–19^ In particular, hexa-substituted benzene ligands have abundant unsaturated bonds to bridge with the square-planar coordinated metal centers of c-MOFs, making it possible to accept/lose electrons, and thus high capacitance can be excepted.^20^ These compelling features have motivated a surge in research activities for 2D c-MOFs for supercapacitors. For instance, Zhao *et al.* used a 2D c-MOFs Cu_3_(HHTP)_2_, as the transparent electrode material that could exhibit an areal capacitance of 939.2 μF cm^−2^.^21^ Nevertheless, these reported 2D c-MOFs can only depend on single redox sites for charge storage, resulting in unsatisfactory capacitance.

Recent studies have demonstrated that dual-redox-site c-MOFs could contribute to high areal capacitance in energy storage devices.^22,23^ However, no studies on bimetallic 2D c-MOFs have been established for transparent MSCs so far, and such a design should be elaborated. However, the interfacial contact resistance between powder crystals may still result in inefficient electron transport. Moreover, the mass loading amount of active material on the film electrode should be small enough to realize ultrahigh transmittance, which involves a trade-off between areal capacitance and transparency. Previous reports indicate that a patterned electrode covers only a small fraction of the surface area, which can be an attractive avenue for significantly increasing the optical transparency of the whole device.^24–26^ As a consequence, further study on the rational construction of 2D c-MOFs, as well as transparent device design, is extremely desirable.

Herein, we construct the dual-metal-sites c-MOF CuNi-HHTP as electrode materials with outstanding capacitance for transparent MSCs. The insertion of Ni^2+^ ion expands the 2D Cu-HHTP to a nanorod-combined CuNi-HHTP, enabling efficient ion/charge transport to active sites for Faraday reactions. Electrochemical mechanism studies, accompanied by density functional theory (DFT) calculations, reveal that Ni-doping can effectively reduce the interfacial resistance and enhance the attraction to pseudocapacitive cations (K^+^) for charge storage. Furthermore, the laser-scribed technique is adopted to make patterned electrodes, which can serve as transparent platforms for coupling the optoelectronic properties of CuNi-HHTP. Impressively, the interdigital CuNi-HHTP MSC exhibits an excellent areal capacitance of up to 28.9 mF cm^−2^ as well as high optical transmittance (over 80%). Our work provides an approach for fabrication of 2D c-MOFs with excellent electrochemical capability, and has far-reaching significance to achieve transparent energy storage devices.

## Results and discussion

**Fig. 1:**
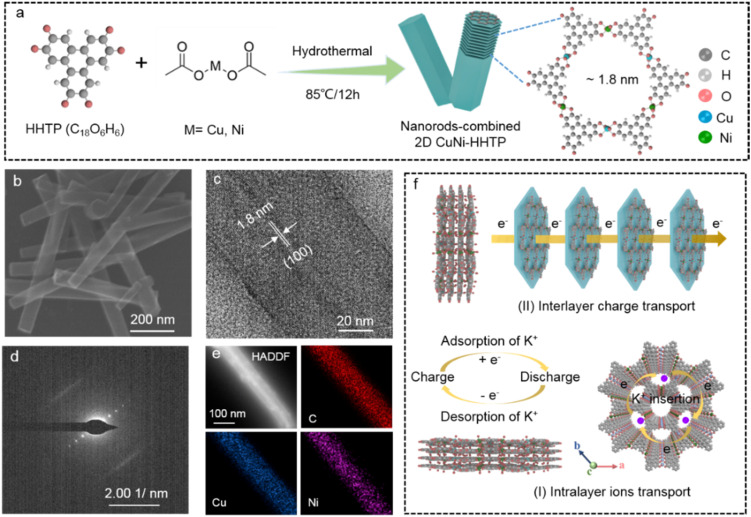
Synthesis illustration, crystal structures, and charge transport models. (a) Synthetic illustration and simulated crystal structure of CuNi-HHTP. (b) SEM, (c) HR-TEM, (d) SAED, and (e) HAADF-STEM and corresponding elemental mapping images (C, Cu, Ni) of CuNi-HHTP. (f) Schematic illustration of ion (intralayer) and charge (interlayer) transport in CuNi-HHTP.

The bimetallic c-MOFs of CuNi-HHTP were fabricated *via* a facile bottom-up synthesis approach (Fig. 1a). Specifically, the Cu/Ni metal ions are linked to oxygen atoms from HHTP ligands on the *ab*-plane to construct a 2D framework with a theoretical pore size of about 1.8 nm, and the hexagonal layers further pack along the *c*-axis in a slipped-parallel ab stacking model to form one-dimensional (1D) channels.^27^ The CuNi-HHTP showcases a particular nanorod-like crystal with a length of about 600 nm and a width of 100 nm (Fig. 1b). The high-resolution transmission electron microscopy (HR-TEM) image in Fig. 1c shows a standard lattice fringe spacing of 1.80 nm, corresponding to an open-window size with arrangement along the (001) direction. The selected area electron diffraction (SAED) pattern shows well-defined diffraction spots (Fig. 1d), which match well with the typical lattice fringes of the (100) plane. The spherical aberration-corrected high-angle annular dark-field scanning TEM (HAADF-STEM) and corresponding elemental mapping images display uniform distribution of C, Cu, and Ni elements throughout the nanorods (Fig. 1e), further confirming the successful synthesis of bimetallic c-MOFs. Typically, CuNi-HHTP has a well-defined porous structure with two types of layers (Fig. 1f): (I) an extended 2D honeycomb framework for insertion of electrolyte ions in the intralayer; and (II) discrete units with permanent 1D channels for charge transport in the interlayer. These unique layers can significantly improve electrical conductivity and create more favourable metal-active sites, thus facilitating electron transfer and redox reaction with enhanced charge-storage efficiency.

**Fig. 2:**
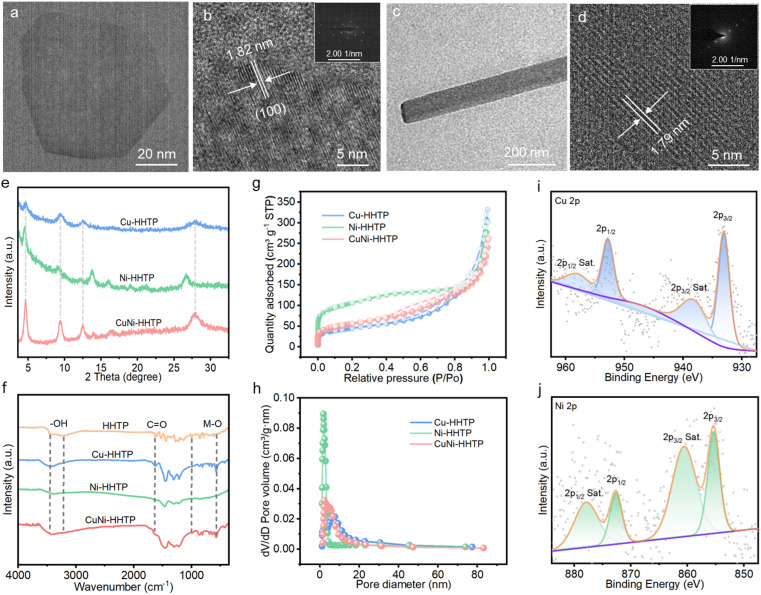
The structure characterizations. (a) TEM and (b) HR-TEM images of Cu-HHTP (inset is SAED). (c) TEM and (d) HR-TEM images of Ni-HHTP (inset is SAED). (e) PXRD patterns, (f) FT-IR spectra, (g) N_2_ adsorption–desorption isotherms, and (h) pore-size distributions of Cu-HHTP, Ni-HHTP and CuNi-HHTP. XPS spectra of (i) Cu 2p and (j) Ni 2p of CuNi-HHTP.

In addition, single-metal Cu-HHTP, Ni-HHTP, and dual-metal CuNi-HHTP with different atomic ratios were fabricated under similar conditions. The Cu-HHTP nanoflakes are aggregated, with an average size of 80 nm (Fig. 2a and S1a†), while the Ni-HHTP has a rod-like shape with a length of about 800 nm and a width of 60 nm (Fig. 2c and S1b†). After incorporation of Ni, the CuNi-HHTP shows a rod-like crystal, with the rod size gradually increasing with increase in the amount of Ni ions, which might be attributed to Ni ions slowing down the rate of crystal growth (Fig. S1c and d†).^28^ The HR-TEM images display a standard lattice fringe spacing of 1.82 nm for Cu-HHTP (Fig. 2b), while a decreased interlayer distance of 1.79 nm is observed for Ni-HHTP, which is along the *c*-axis or parallel to the 1D channels (Fig. 2d).^29^ The SAED images are consistent with the *c*-axis orientation and hexagonal structure of previous reports,^21^ further confirming the high crystallinity (Fig. 2b and d insets). The crystalline structure of the as-synthesized samples was analysed using powder X-ray diffraction (PXRD). As shown in Fig. 2e, the characteristic peaks at 4.7, 9.5, 12.5, and 28.1 are indexed to the (100), (200), (210), and (001) lattice planes of layered structures, respectively.^30^ Notably, the diffraction peak of the (100) plane shifts towards a higher angle direction, which matches well with the decreased lattice distances due to doping of Ni into the Cu-HHTP framework. The Fourier transform infrared spectrum (FT-IR) was recorded to demonstrate the coordination features of the chemical bonds in the c-MOFs. As shown in Fig. 2f, the vibration peak of –OH around 3500 cm^−1^ has disappeared, indicating successful transformation of –OH to C–O. The peaks at 1635 and 1455 cm^−1^ can be attributed to the unsaturated bonds of the organic ligands. Meanwhile, the absorption bands in the range of 500 to 550 cm^−1^ are attributed to Cu/Ni stretching vibrations, further verifying the existence of coordination between oxygen and metal ions in CuNi-HHTP.^31^

In addition, the porosity of CuNi-HHTP was examined using N_2_ adsorption–desorption isotherm (Fig. 2g and h), and was identified as a mixed I/IV type with inherent micropores along with mesopores.^32^ The Brunauer–Emmett–Teller (BET) surface areas and pore volumes of different c-MOFs are presented in Table S1.† After incorporation of Ni ions, the BET surface of CuNi-HHTP increased to 192.1 m^2^ g^−1^. This porous structure with a larger specific surface area and more rich pores will facilitate electron and ion transport to active sites for the redox reaction. X-ray photoelectron spectroscopy (XPS) was then used to clarify the chemical bonds and the state of the elements in CuNi-HHTP. As depicted in Fig. 2i and j, the peaks at 952.9 (2p_1/2_) and 933.1 eV (2p_3/2_), as well as the satellite peaks at 958.4 and 938.8 eV, in the high-resolution Cu 2p XPS spectrum can be assigned to the bonding of Cu^2+^ species, while the peaks at 872.7 and 855.4 eV and the satellite peaks at 877.9 and 860.6 eV all belong to Ni^2+^ species.^33^ These results confirm that the valence state of the metal elements in CuNi-HHTP is +2, and is therefore expected to participate in redox reactions.

**Fig. 3:**
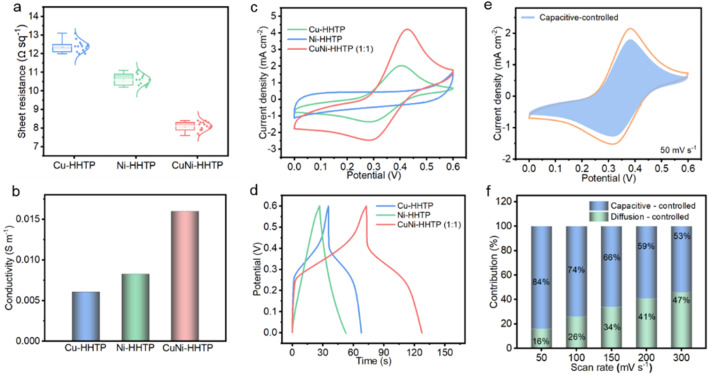
The electrochemical performances. (a) Sheet resistances, (b) electrical conductivities, (c) CV curves, and (d) GCD curves of Cu-HHTP, Ni-HHTP, and CuNi-HHTP electrodes. (e) CV with capacitive-controlled contribution at 50 mV s^−1^. (f) Contribution ratios of capacitive- and diffusion-controlled currents for the CuNi-HHTP electrode.

Compared with Cu-HHTP, the dual-metal-sites CuNi-HHTP can greatly reduce its intrinsic resistance and enhance electron transfer through 1D channels at the electrode/electrolyte interface. As shown in Fig. 3a, the lowest sheet resistance (*R*_s_) of CuNi-HHTP (8.0 Ω sq^−1^) indicates a fast electron transfer rate, owing to the reduced transport distance for the CuNi-HHTP nanorods. Furthermore, the electrical conductivity of CuNi-HHTP is found to be 1.6 × 10^−2^ S m^−1^ (Fig. 3b), which is much higher than that of Cu-HHTP (6.1 × 10^−3^ S m^−1^) and Ni-HHTP (8.3 × 10^−3^ S m^−1^). These findings suggest that the difference in interface resistance of c-MOFs stems from electron transport between 1D channels and 2D framework structures.

To determine the effect of metal species on the electroactive nature of the different c-MOFs, cyclic voltammetry (CV) was conducted in 3 M KCl using a three-electrode system.

As shown in Fig. 3c, the CuNi-HHTP electrode exhibits obvious redox peaks, indicating that the pseudo-capacitance mainly arises from faradaic reactions. The improved current density suggests that the integration of Cu and Ni ions in CuNi-HHTP has a positive effect on electrochemical behaviour. As expected, galvanostatic charge–discharge (GCD) curves show that the CuNi-HHTP presents the longest charge–discharge time, compared with Cu-HHTP and Ni-HHTP (Fig. 3d), revealing the best charge-storage capability. Furthermore, CV curves at different scan rates and approximately symmetric GCD curves shown in Fig. S2† demonstrate the excellent pseudocapacitive characteristic of CuNi-HHTP. The percentage of capacitive contribution was subsequently analysed based on the following equation:

1 *i* = *k*_1_*v* + *k*_2_*v*^1/2^

where *k*_1_*v* and *k*_2_*v*^1/2^ represent the current contributed by capacitive- and diffusion-controlled behaviour, respectively.^34^ The capacitive-controlled contribution accounts for 84% at a low scan rate, suggesting good capacitive behaviour (Fig. 3e and f). Based on the optimized conditions, the CuNi-HHTP electrode with a Cu/Ni atomic ratio of 1 : 1 has the highest areal capacitance (Fig. S3†), indicating that the intrinsically synergistic effect between Cu and Ni, together with the appropriate mass ratio, is responsible for the increased specific capacitance.

**Fig. 4:**
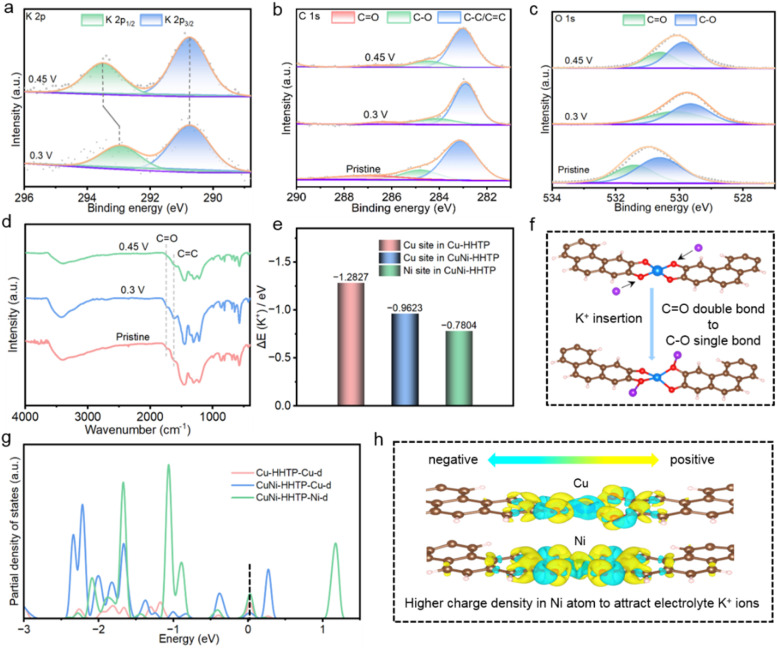
Mechanism study and structural evolution of the CuNi-HHTP electrode during the charge-storage process in KCl-based electrolyte. *Ex situ* XPS spectra of (a) K 2p, (b) C 1s, (c) O 1s. (d) FT-IR spectra of the CuNi-HHTP electrode at different charging states. (e) DFT-calculated Δ*E*_k+_ of Cu-HHTP and CuNi-HHTP. (f) Illustration of K^+^ storage process. (g) Partial density of states and (h) the electron density difference between Cu and Ni metal centers in CuNi-HHTP.

To reveal the charge-storage mechanism of CuNi-HHTP in KCl-based electrolyte, *ex situ* XPS spectra were recorded to trace the structural and electronic evolution at different charging states. Fig. 4a shows the high-resolution core-level K 2p XPS, which can be deconvoluted into two components at 293.1 and 290.8 eV, attributed to the configuration of K 2p_1/2_ and K 2p_3/2_, respectively. The enhanced intensity of K^+^ can be seen with increase of the charge voltage from 0.3 V to 0.45 V; meanwhile, the K 2p_1/2_ peak shifts to a higher binding energy, revealing an obvious K^+^ storage process. There are characteristic peaks in Fig. 4b in the C 1s XPS at 282.9, 284.5, and 286.3 eV, which can be indexed to the three different bonding species of C=C/C–C, C–O, and C=O, respectively. Meanwhile, the intensity of the C=O bond peak experiences a decreasing trend and the intensity of the C–O bond peak an increase, with a potential increase from 0.3 V to 0.45 V, suggesting transformation of the C=O double bond to a C–O single bond induced by the insertion of K^+^ ions.^35^Fig. 4c shows the high-resolution O 1s spectrum, and the same vibration trends for the C–O and C=O bonds can be seen as for the C 1s spectrum. Furthermore, *ex situ* FT-IR spectra were collected to elucidate the redox mechanism of CuNi-HHTP at different charging stages. As shown in Fig. 4d, the intensity of the C=O peak decreases during the charging process, providing evidence that the C=O double bond is broken during the redox reaction.^36^

DFT calculations were performed to unveil the effect of the Ni ion on the electronic properties and adsorption sites of K^+^ electrolyte on the surface of CuNi-HHTP. Fig. 4e shows the adsorption energy of K^+^ (Δ*E*_k+_) ions on Cu-HHTP and CuNi-HHTP materials. The calculated results reveal that the Δ*E*_k+_ values are −0.7804 and −0.9623 eV for the Ni sites and the Cu sites of CuNi-HHTP, respectively, while the value is −1.2827 eV for Cu-HHTP, suggesting that Ni incorporation is favourable for enhanced K^+^ adsorption and ion transport kinetics.^37^ The charge-storage mechanism of CuNi-HHTP can be illustrated as in Fig. 4f and S4,† validating that faradaic reactions occur with transformation of the C=O double bond to a C–O single bond due to insertion/extraction of K^+^ ions. Fig. 4g illustrates the partial density of states (DOS), the DOS increases significantly after Ni-doping, particularly in the Ni d orbital of CuNi-HHTP, indicating more available electronic states near the Fermi level (around 0 eV).^38,39^ The charge density difference between Cu and Ni sites in CuNi-HHTP was further evaluated. As depicted in Fig. 4h, the charge density in the Ni atom is much higher than that of the Cu atom, indicating that Ni has stronger electronic capability and is more likely to attract electrolyte K^+^ ions onto the electrode surface, facilitating electron transport during the charge-storage process.

**Fig. 5:**
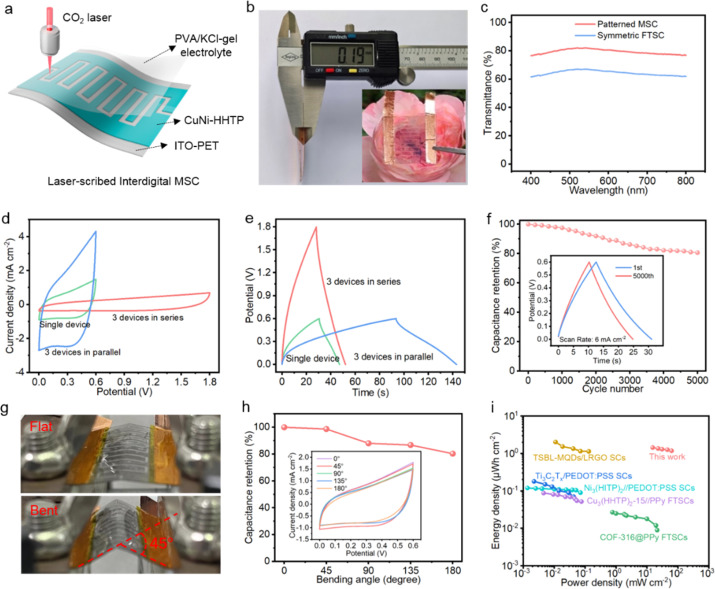
Optoelectronic behaviours of CuNi-HHTP devices. (a) Illustration of laser-scribed interdigital MSC. (b) Thickness of the MSC (inset is a digital photograph). (c) Comparison of optical transmittance for the two devices. (d) CV curves and (e) GCD curves of a single device and three MSC devices connected in series and in parallel. (f) Cycling stability. (Inset) The first and 5000th GCD curves at a current density of 6 mA cm^−2^. (g) Optical photographs of the MSC in flat and bent states. (h) Capacitance retention under different bending angles from 0° to 180°. (Inset) CV curves in the bent states. (i) Ragone plots of the CuNi-HHTP MSC in comparison with those of previously reported transparent devices.

In addition to improved electrochemical behaviour, optical transparency also plays a significant role in the corresponding device design. Taking advantage of the laser-scribing technique, the interdigital MSC device can effectively improve the optical transmittance while ensuring the amount of active materials present. After exposure to a CO_2_ laser, an in-plane patterned electrode is obtained (Fig. S5†), revealing a figure width of about 2 mm and an inter-figure space of 500 μm. The Cu and Ni elements are uniformly distributed on the electrode surface, proving that a high mass loading of CuNi-HHTP is achieved by adopting the patterned design. Two types of energy storage device were fabricated based on CuNi-HHTP, namely a symmetric sandwich-type flexible transparent supercapacitor (FTSC, see ESI Fig. S6†) and an interdigital MSC (Fig. 5a). It is noted that the thickness of the MSC is only 190 μm (Fig. 5b), which is much thinner than the FTSC (370 μm). The optical transmittance of the patterned MSC at a wavelength of 550 nm is over 80% (Fig. 5c), while for the FTSC it is 65.2%. The shape of the CV curves can be substantially maintained with increase in scan rates from 50 to 300 mV s^−1^ (Fig. S7a and c†), enabling excellent rate capability as well as fast charge-storage capacity. According to the GCD curves (Fig. S7b and d†), the MSC exhibits a maximum areal capacitance of 28.94 mF cm^−2^ at a current density of 50 μA cm^−2^ (Fig. S7e†), which is almost three times that of the FTSC (10.44 mF cm^−2^). Electrochemical impedance spectra (EIS) for the two devices were then recorded to investigate the electrochemical reaction kinetics. The equivalent circuits are illustrated in Fig. S7f,† which involve the interface resistance (*R*), the charge transfer resistance (*R*_ct_), the Warburg diffusion resistance (*W*), the electrical double-layer capacitance (*C*_EDLC_), and the pseudocapacitance (*C*_p_). The semicircles in high-frequency regions represent *R*_ct_ and the straight lines in low-frequency regions are related to ion diffusion resistance. The smaller *R*_ct_ of the MSC device suggests faster reaction kinetic.

The excellent electrochemical performance of CuNi-HHTP inspired us to evaluate the possibility of practical applications. Three identical MSC devices were connected in series and in parallel for the demands of higher capacitance and output voltage. As depicted in Fig. 5d, the series configuration extends the voltage window up to 1.8 V, while the discharge time is three times that of a single device (Fig. 5e), demonstrating excellent scalability and uniformity of the MSCs. Fig. S8† illustrates three series-connected devices that work as a power supply to successfully operate a red light-emitting diode for more than 60 s, which can be conformal with other flexible transparent electronics. Long cycling stability has also been tested at a current density of 6 mA cm^−2^, with 95% of the initial capacitance kept after 1000 cycles of charging and discharging, and the capacity retention of MSC was maintained at 80% after 5000 cycles (Fig. 5f and inset). The structure of CuNi-HHTP was further characterized after the cycling (Fig. S9†), which implied that the host structure was maintained with only a slight position change, thus enabling good stability.

The mechanical flexibility of the patterned MSC was measured by a bending test, as presented in Fig. 5g. The CuNi-HHTP MSC can maintain 80% of its capacitance value under various harsh mechanical bending tests, from 0° to 180° (Fig. 5h and inset). The energy and power densities of the MSC based on CuNi-HHTP were further compared with those of other transparent devices (Fig. 5i). More details about transmittance, areal capacitance, energy density, and power density are shown in Table S2.† Remarkably, both the optical transmittance and areal capacitance of our MSC were superior to these recently proposed devices. The CuNi-HHTP MSC shows an extremely high volumetric energy density of 1.45 μW h cm^−2^ with corresponding power density of 61.38 mW cm^−2^, which is much higher than for the recently emerged transparent devices based on Cu_3_(HHTP)_2_-15//PPy,^21^ COF-316@PPy,^40^ MXene QDs/Graphene,^41^ and Ti_3_C_2_T_*x*_/PEDOT:PSS,^42^ and is even comparable to that of solid-state MSCs based on c-MOFs, such as LSG/Ni-CAT.^29^

## Conclusions

We have demonstrated 2D c-MOFs dual-metal-sites CuNi-HHTP as high-capacitance electrode materials for transparent MSCs. The customized Ni ion incorporation enables nanorod-combined CuNi-HHTP with efficient ion and electron transport at the electrode/electrolyte interface. With the support of electrochemical mechanism studies and DFT calculations, the extraordinary high capacitance of CuNi-HHTP can be attributed to faradaic reactions occurring on the dual-metal sites with the insertion/extraction of K^+^ ions. Moreover, by adopting a laser-scribing strategy, the interdigital CuNi-HHTP MSC delivers excellent optical transmittance of over 80%, ultrahigh areal capacitance of up to 28.9 mF cm^−2^, and energy density of 1.45 μW cm^−2^, and a power density of 61.38 mW cm^−2^. This work highlights the importance of constructing 2D c-MOFs, and promotes the development of flexible transparent electronics.

## Data availability

Additional experimental data supporting this article are included in the ESI.†

## Author contributions

C. Zhao designed the research. S. K. Wang performed c-MOFs synthesis and characterization. S. Q. Chen performed patterned device. B. Han performed electrode characterization. S. H. Wan and Q. J. Bai performed electrochemical measurements. M. A. Hu performed DFT calculations. F. Y. Kang assisted with mechanism analysis. All authors contributed to the final version of the manuscript.

## Conflicts of interest

There are no conflicts to declare.

## Supplementary Material

SC-OLF-D5SC01584G-s001
