# Supplementary material for: Dual-metal sites enable conductive metal–organic frameworks with extraordinary high capacitance for transparent energy storage devices
Source: Chem Sci. 2025 Apr 15;16(21):9276–83. doi: 10.1039/d5sc01584g (PMC12020011; doi:10.1039/d5sc01584g)
Supplement: SC-016-D5SC01584G-s001 [file SC-016-D5SC01584G-s001.pdf]

Supplementary Information (SI) for Chemical Science.
This journal is © The Royal Society of Chemistry 2025

Supporting Information

Dual-metal-sites enable conductive metal-organic frameworks with extraordinary high capacitance for transparent energy storage devices

Cui-e Zhao [a], Shuaikang Wang [a], Shaoqiang Chen [a], Bin Han [a], Shouhao Wan [a], Qijia Bai [a], Mingao Hu,[a] Fangyuan Kang,[b] Ruiqing Liu [a*], Jiahui Li [a*], Yanwen Ma [a, c*] and Qichun Zhang [b*]

[a]State Key Laboratory of Flexible Electronics (LoFE) & Institute of Advanced Materials (IAM), Nanjing University of Posts and Telecommunications, 9 Wenyuan Road, Nanjing, Jiangsu 210023, China

[b]Department of Materials Science and Engineering, City University of Hong Kong, Hong Kong SAR999077, P. R. China

[c]Suzhou Vocational Institute of Industrial Technology, 1 Zhineng Avenue, Suzhou International Education Park, Suzhou 215104, China.

Contents

1. Experimental Procedures
2. Characterization
3. Devices fabrication
4. Electrochemical Measurements
5. Calculations
6. Figure S1-S9, Table S1, Table S2

1. Experimental Procedures

Materials: All chemicals were purchased commercially and used without further purification. Copper (II) acetate monohydrate and nickel (II) acetate tetrahydrate were purchased from Aladdin Chemicals Reagent Co., Ltd. (Shanghai, China). 2,3,6,7,10,11-hexahydroxytriphenylene hydrate (HHTP, ≥95%) was purchased from Macklin Chemical Reagent Co., Ltd. (Shanghai, China).

Preparation of CuNi-HHTP: The CuNi-HHTP nanorods was prepared through a facile hydrothermal method, following a modified procedure from previous report.[1] $Cu(CO_2CH_3)_2·H_2O$ (0.3 mM), $Ni(CO_2CH_3)_2·4H_2O$ (0.3 mM) and HHTP (0.3 mM) were dissolved in a mixture of 5 mL deionized water and 5 mL DMF under sonication for 30 min in room temperature (RT) until the color became dark blue. Then, the mixture was heated at 85 °C for 10 h. The final product was washed with ultrapure water and acetone thoroughly, and then dried at 60 °C under vacuum for overnight. For comparison, the CuNi-HHTP with selected atomic ratios of Cu/Ni (1:1, 1:3 and 3:1) were synthesized, which were denoted as CuNi-HHTP (1:1), CuNi-HHTP (1:3) and CuNi-HHTP (3:1), respectively.

The Cu-HHTP and Ni-HHTP were prepared under the similar conditions as that of CuNi-HHTP. For the preparation of Cu-HHTP, 0.6 mM $Cu(CO_2CH_3)_2·H_2O$ and 0.3 mM HHTP were dissolved in a mixed solution of 5 mL deionized water and 5 mL DMF, which was sonicated at RT for 30 min, then, heated at 85°C for 10 h and dried at 60 °C under vacuum for overnight. Similarly, as for the Ni-HHTP, 0.6 mM $Ni(CO_2CH_3)_2·4H_2O$ and 0.3 mM HHTP were dissolved in 20 mL deionized water, sonicated at RT for 30 min, then, heated at 85°C for 10 h and dried at 60 °C under vacuum for overnight.

2. Characterizations

The morphology and structure of the fabricated samples were characterized by field emission scanning electron microscope (FE-SEM, Hitach S4800), transmission electron microscopy (TEM), high-resolution transmission electron microscopy (HR-TEM, FEI Talos F200X). X-ray diffraction (XRD) patterns were measured on a Bruker diffractometer (D8 Advance A25) with Cu Kα radiation (λ=1.5Å). Brunauer-Emmett-Teller (BET) surface area and pore size distributions were carried out by a N_2 absorption/desorption analyzer (ASAP 2020 Plus HD88, Micrometrics). X-ray photoelectron spectroscopy (XPS) analysis was detected by a photoelectron spectrometer (Axis Supra). The transmittance was tested using Ultraviolet-visible Spectrophotometer (UV, PerkinElmer LAMBDA 35). The thickness of film electrodes was detected by a step profiler (Bruker, DektakXT Stylus Profiler).

3. Devices fabrication

Fabrication of Laser-scribed interdigital micro-supercapacitor (MSC): Before assembling, the transparent CuNi-HHTP thin film electrode was fabricated as follows: the CuNi–HHTP solution (1 mg mL^{-1}) were sprayed onto the surface of the ITO-PET substrate (3 × 3 cm^2), which had already been treated by O_2 plasma to allow the CuNi-HHTP layer to attach firmly onto the substrate. The loading mass of the CuNi-HHTP electrode is 0.75 mg cm^{-2}.

Subsequently, a well-established CO_2 laser-scribing technique (Universal VLS 6.75 Laser Platform System, laser light spot diameter of 100 μm Approx., power 18%, speed 1.27 m/s, laser optics focal length 50 mm) was adopted for the patterned CuNi–HHTP electrode design (10 interdigital fingers, width of 2 mm, length of 9.5 mm and interspace of 500 μm). Afterwards, Cu current collectors were taped around the edges of patterned electrode, following by the PVA/KCl gel electrolyte (0.2 mL) carefully drop-casted (repeated two times) onto the patterned regions and solidified overnight. Finally, the interdigitated transparent MSC device was assembled.

The PVA/KCl gel electrolyte was prepared as follows: 2 g of PVA powder and 7.5 g of KCl were dissolved in 20 mL of deionized water and stirred vigorously at 85 °C until a clear transparent solution was formed.

Fabrication of sandwich-type flexible transparent supercapacitor (FTSC): The process of fabricating the sandwich-type symmetrical FTSC device was as follows: two identical pieces of CuNi–HHTP thin film electrodes (3 × 3 cm^2) were assembled into a sandwich structure with PVA/KCl gel as solid-state electrolyte. After that, the FTSC device was allowed to dry under ambient conditions for 2 h to allow better penetration of electrolyte into electrodes.

4. Electrochemical Measurements

All electrochemical measurements were performed on a workstation (Bio-logic, VMP3). The electrochemical characteristics of the CuNi-HHTP electrode were conducted in a three-electrode system with liquid electrolyte or in a two-electrode system with solid-state electrolyte, using Ag/AgCl and Pt wire as the reference electrode and the counter electrode, respectively. The sheet resistance was detected by a Four-probe Tester (HP504).The electrical conductivity of c-MOFs powder pellets was measured with a four-point probe method using Keithley 4200. The pellets of c-MOFs were pressed at a pressure of ≈1 GPa. The Nyquist plots were studied in a frequency range of 0.01 to 100 000 Hz.

5. Energy and power density Calculations:

The C_A was calculated by GCD curves with the equation (1):

$$C_A = \frac{It}{SV} \tag{1}$$

where *I* is discharging current, *V* is voltage window after deducting the IR drop, *S* is valid area of the electrodes or the devices, *t* is discharging time.

The energy (*E*) and power (*P*) densities were obtained from the following equations (3) and (4):

$$E = \frac{1}{2} \times C_A \times \frac{(V)^2}{3600} \tag{2}$$

$$P = \frac{E}{t} \times 3600 \tag{3}$$

The electric conductivity (*σ*) was tested based on the thickness (*d*) and resistance (*R*) by the following equation (5):

$$\sigma = \frac{1}{Rd} \tag{4}$$

6. DFT Calculations:

All calculations were performed using density functional theory (DFT) implemented in the Vienna Ab initio Simulation Package (VASP).[2-4] Starting from the crystal structure of Cu-HHTP, the atomic model of CuNi-HHTP was constructed by partially substituting metal atoms. Geometry optimizations were conducted with the PBEsol functional,[5] combining with the projector augmented wave (PAW) pseudopotential approach for electron interactions and scalar relativistic effects.[6] The plane-wave cutoff energy was set to 500 eV (ENCUT = 500), with an electronic convergence criterion of 10^{-5} eV (EDIFF = $1E^{-5}$) and an ionic force convergence criterion of 0.01 eV/Å (EDIFFG = -0.01). The Brillouin zone was sampled using a Monkhorst-Pack grid, with a k-point density of 2×2×1 for geometry optimizations and 4×4×1 for static energy calculations. A vacuum layer of 25 Å was applied along the vertical (z-axis) direction to prevent interlayer interactions. To simulate the solvent effect of the KCl electrolyte environment, an implicit solvation was considered using the VASPSol model.[7] The dielectric constant (ε) was set to 78.4 for water. The adsorption energy (E_{ads}) was calculated using the following formula:

$$E_{ads} = E_{surface + ion} - E_{surface} - E_{ion}$$

The energy of the isolated ion (E_{ion}) was computed within a large vacuum box of 20 Å×20 Å ×25 Å. Static calculations were performed with a stricter electronic convergence criterion of EDIFF=$1E^{-6}$ to improve the accuracy of adsorption energy determinations.

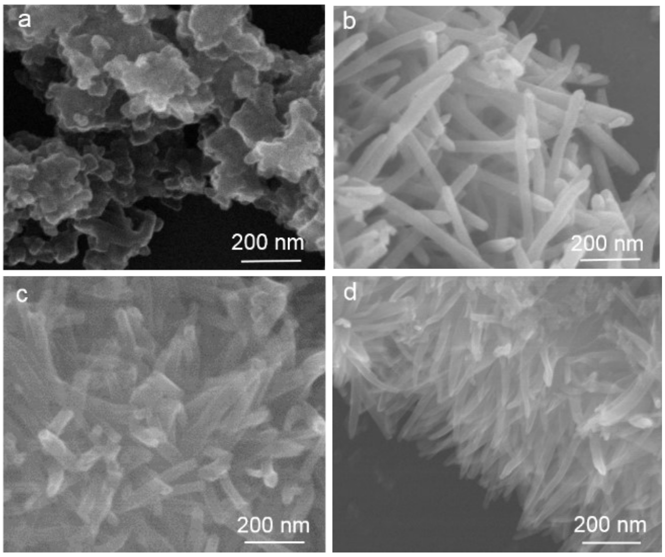

Fig. S1 SEM images of (a) the Cu-HHTP, (b) the Ni-HHTP, and CuNi-HHTP with different Cu/Ni atomic ratios (c) CuNi-HHTP (3:1), (d) CuNi-HHTP (1:3).

Table S1 Comparison of BET surface area, pore size, and pore volume of different samples.

**Samples**	**Cu-HHTP**	**Ni-HHTP**	**CuNi-HHTP**
BET surface area (m^2g^{-1})	153.82	355.11	192.07
BJH average pore size (nm)	12.909	7.076	8.692
BJH volume (cm^3g^{-1})	0.5188	0.4072	0.4727

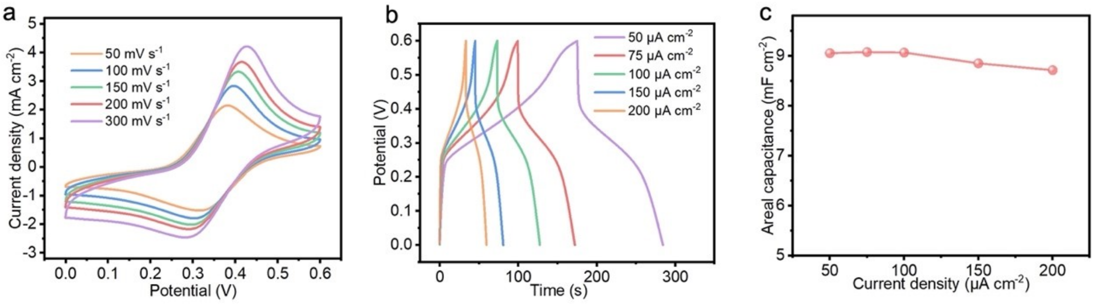

Fig. S2 The electrochemical performances of CuNi-HHTP electrodes. (a) CV curves at scan rates from 50 to 300 mV s^{-1}, (b) GCD curves at current densities from 50 to 200 μA cm^{-2}, (c) Area capacitances calculated from GCD profiles of the CuNi-HHTP electrode.

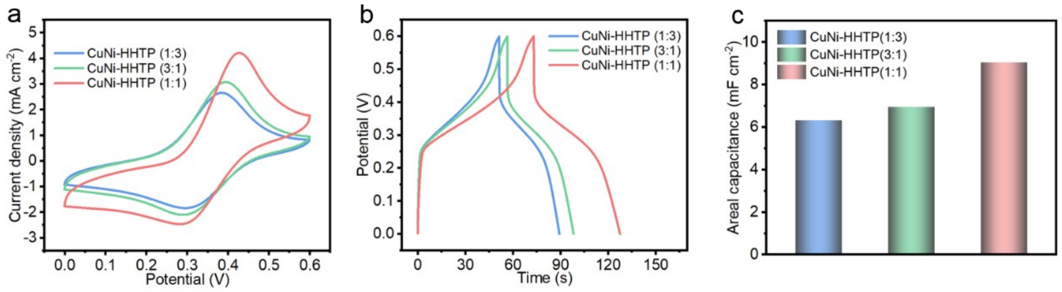

Fig. S3 The electrochemical performances of CuNi-HHTP electrodes with different Cu/Ni atomic ratios. (a) CV curves at scan rate of 100 mV s^{-1}; (b) GCD curves at current density of 100 μA cm^{-2}; (c) Areal capacitances by the GCD profiles.

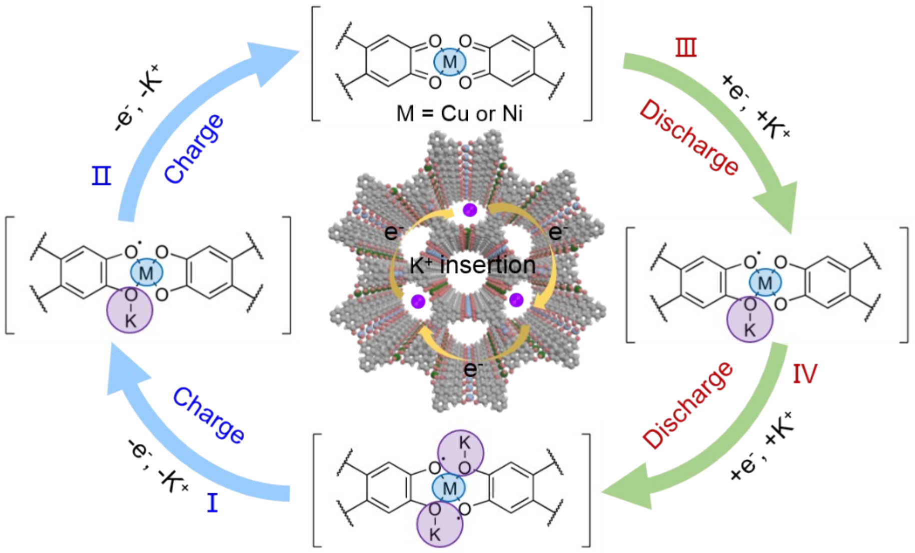

Fig. S4 The schematic diagram of K^+ charge storage during the charge/discharge process. The binding sites and variation of valence states are marked by circles.

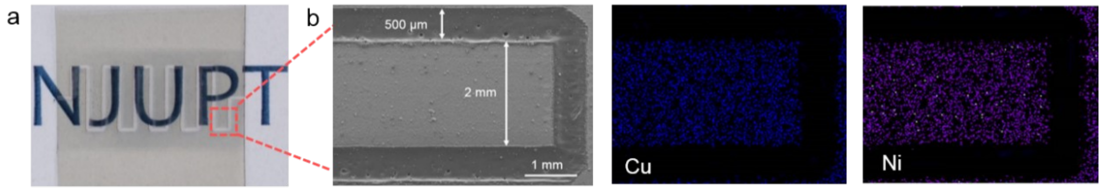

Fig. S5 (a) Digital photograph, (b) SEM image and element characterization (Cu, Ni) of laser-scribed CuNi-HHTP electrode.

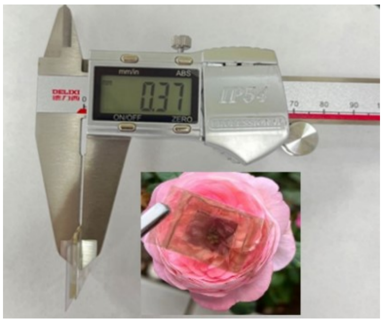

Fig. S6 The thicknesses of the sandwich-type FTSC (inset is the photograph of the device)

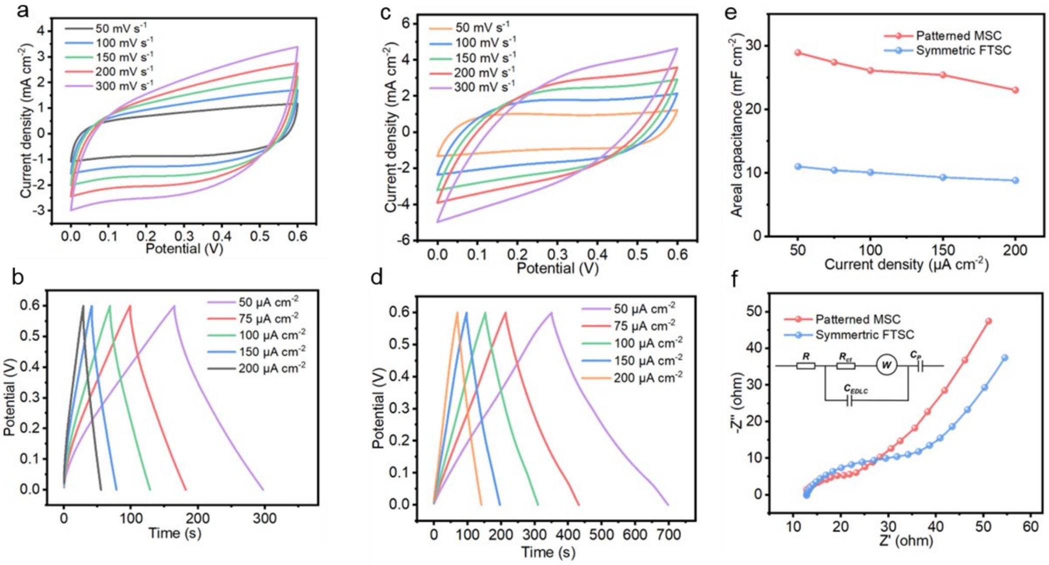

Fig. S7 (a) CV curves and (b) GCD curves of the sandwich-type FTSC device, (c) CV curves and (d) GCD curves of the patterned MSC device, (e) Areal capacitances comparison of the two devices by the GCD profiles, (f) Nyquist plots of the MSC and the FTSC devices (inset is the equivalent circuit of the devices, *R* is the interface resistances; R_{ct} is charge transfer resistance; *W* is Warburg diffusion resistance; C_{EDLC} is electrical double-layer capacitance; C_P is the pseudocapacitance).

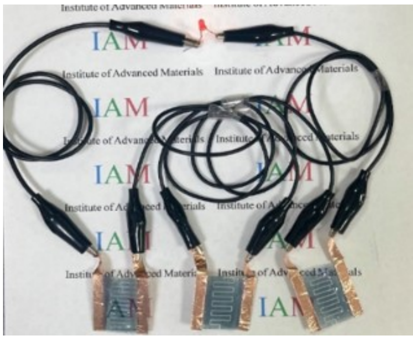

Fig. S8 Demonstration of the LED driven by three CuNi-HHTP MSC devices connected in series.

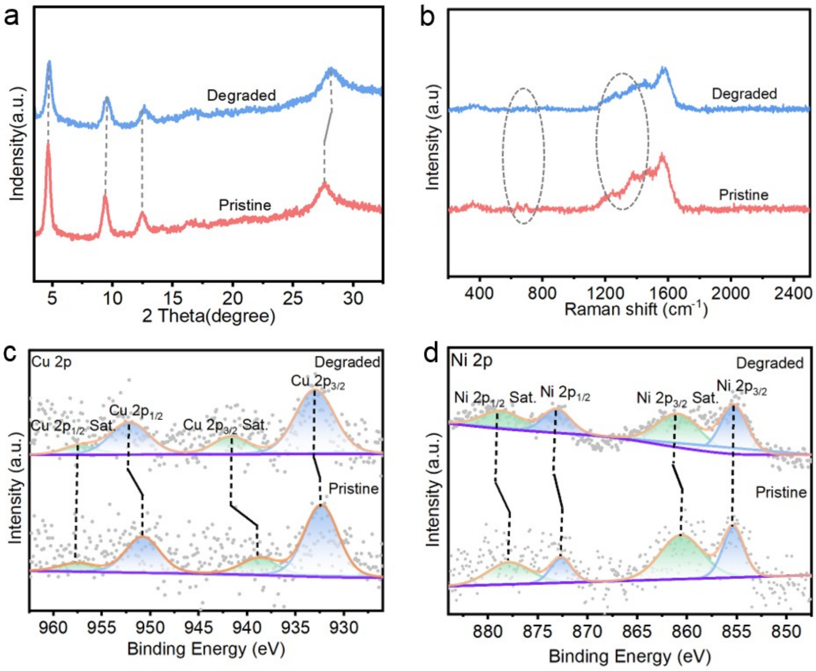

Fig. S9 Structure analysis of the CuNi-HHTP material. (a) PXRD patterns, (b) Raman spectra, XPS spectra of (c) Cu2p and (d) Ni2p of the CuNi-HHTP in the pristine and 5000th charged states at a current density of 6 mA cm^{-2}.

Table S2 Performance of (transparent) supercapacitors based on conductive MOFs and 2D materials.

Active materials	Device design	Transmittance (%)	Areal capacitance (mF cm^{-2})	Power density (mW cm^{-2})	Energy density (μWh cm^{-2})	Ref
COF-316@PPy	COF-316@PPy symmetric FTSCs	53	0.783	21	0.027	8
$Cu_3(HHTP)_2$// PPy	$Cu_3(HHTP)_2$-15// PPy asymmetric FTSCs	65	0.7844	0.075	0.088	9
$Ni_3(HITP)_2$// PEDOT:PSS	$Ni_3(HITP)_2$// PEDOT:PSS asymmetric SCs	61	1.06	0.07	0.12	10
$Ti_3C_2T_x$	$Ti_3C_2T_x$/SWCNT asymmetric SCs	72	1.6	0.025	0.05	11
$Ti_3C_2T_x$/ PEDOT:PSS	$Ti_3C_2T_x$/PEDOT: PSS SCs	61.6	3.1	0.042	0.07	12
TSBL-MQDs/LRGO	TSBL-QDs/ LRGO SCs	80	16.17	0.1294	2.04	13
PEDOT:PSS/ MoO_3-x nanolayers	PEDOT:PSS/ MoO_3/SA devices	＞70	2.99	-	-	14
AgNWs-MoS_2	TFE-TMSCs	77.5	27.6	1.472	2.453	15
Multilayer graphene@Ag nanowires	TFSCs	65	0.3	$2.5*10^{-3}$	$9.7*10^{-3}$	16
Cu-CAT-NWAs	Cu–CAT NWA SC	-	0.022	2.6 Whkg^{-1}	0.2 kWkg^{-1}	17
$Ni_2[CuPc(NH)_8]$/ EG	$Ni_2[CuPc(NH)_8]$/ EG-2 MSCs	-	18.9	168	1.7	18
LSG/Ni-CAT	LSG/Ni-CAT MSCs	-	15.2	7.0	4.1	19
CuNi-HHTP nanorods	CuNi-HHTP MSCs	82	28.94	61.38	1.45	This Work

Supporting References

1. M. Hmadeh, Z. Lu, Z. Liu, F. Gándara, H. Furukawa, S. Wan, V. Augustyn, R. Chang, L. Liao, F. Zhou, E. Perre, V. Ozolins, K. Suenaga, X. Duan, B. Dunn, Y. Yamamto, O. Terasaki and O. M. Yaghi, *Chem. Mater.*, 2012, **24**, 3511-3513.
2. W. Kohn and L. J. Sham, *Phys. Rev.*, 1965, **140**, A1133-A1138.
3. G. Kresse, Furthmüller, *J. Phys. Rev. B*, 1996, **54**, 11169.
4. G. Kresse and D. Joubert, *Phys. Rev. B*, 1999, **59**, 1758-1775.
5. J. P. Perdew, A. Ruzsinszky, G. I. Csonka, O. A. Vydrov, G. E. Scuseria, L. A. Constantin, X. Zhou and K. Burke, *Phys. Rev. Lett.*, 2008, **100**, 136406.
6. P. E. Blöchl, *Phys. Rev. B*, 1994, **50**, 17953-17979.
7. K. Mathew, R. Sundararaman, K. Letchworth-Weaver, T. A. Arias and R. G. Hennig, *J. Chem. Phys.*, 2014, **140**, 084106.
8. W. Wang, W. Zhao, T. Chen, Y. Bai, H. Xu, M. Jiang, S. Liu, W. Huang and Q. Zhao, *Adv. Funct. Mater.*, 2021, **31**, 2010306.
9. W. Zhao, T. Chen, W. Wang, S. Bi, M. Jiang, K. Y. Zhang, S. Liu, W. Huang and Q. Zhao, *Adv. Mater. Interfaces*, 2021, **8**, 2100308.
10. W. Zhao, T. Chen, W. Wang, B. Jin, J. Peng, S. Bi, M. Jiang, S. Liu, Q. Zhao and W. Huang, *Sci. Bull.*, 2020, **65**, 1803-1811.
11. C. Zhang, B. Anasori, A. Seral Ascaso, S. H. Park, N. McEvoy, A. Shmeliov, G. S. Duesberg, J. N. Coleman, Y. Gogotsi and V. Nicolosi, *Adv. Mater.*, 2017, **29**, 1702678.
12. S. Ren, X. Pan, Y. Zhang, J. Xu, Z. Liu, X. Zhang, X. Li, X. Gao, Y. Zhong, S. Chen and S. D. Wang, *Small*, 2024, **20**, 2401346.
13. Y. Yuan, L. Jiang, X. Li, P. Zuo, X. Zhang, Y. Lian, Y. Ma, M. Liang, Y. Zhao and L. Qu, *Adv. Mater.*, 2022, **34**, 2110013.
14. M. Yoonessi, A. Borenstein, M. F. El-Kady, C. L. Turner, H. Wang, A. Z. Stieg and L. Pilon, *ACS Appl. Energy Mater.,* 2019, **2**, 4629-4639.
15. J. Li, Q. Shi, Y. Shao, C. Hou, Y. Li, Q. Zhang and H. Wang, *Energy Storage Mater.*, 2019, **16**, 212-219.
16. Y. Zhong, X. Zhang, Y. He, H. Peng, G. Wang and G. Xin, *Adv. Funct. Mater.*, 2018, **28**, 1801998.
17. W. H. Li, K. Ding, H. R. Tian, M. S. Yao, B. Nath, W. H. Deng, Y. Wang and G. Xu, *Adv. Funct. Mater.*, 2017, **27**, 1702067.
18. M. Wang, H. Shi, P. Zhang, Z. Liao, M. Wang, H. Zhong, F. Schwotzer, A. S. Nia, E. Zschech, S. Zhou, S. Kaskel, R. Dong and X. Feng, *Adv. Funct. Mater.*, 2020, **30**, 2004410.
19. H. Wu, W. Zhang, S. Kandambeth, O. Shekhah, M. Eddaoudi and H. N. Alshareef, *Adv. Energy Mater.*, 2019, **9**, 1900482.
